# Patterns of HIV/SIV Prevention and Control by Passive Antibody Immunization

**DOI:** 10.3389/fmicb.2016.01739

**Published:** 2016-11-02

**Authors:** Hiroyuki Yamamoto, Tetsuro Matano

**Affiliations:** ^1^AIDS Research Center, National Institute of Infectious DiseasesTokyo, Japan; ^2^Department of AIDS Vaccine, The Institute of Medical Science, The University of TokyoTokyo, Japan

**Keywords:** HIV, SIV, neutralizing antibodies, passive immunization, CD8^+^ T cells

## Abstract

Neutralizing antibody (NAb) responses are promising immune effectors for control of human immunodeficiency virus (HIV) infection. Protective activity and mechanisms of immunodeficiency virus-specific NAbs have been increasingly scrutinized in animals infected with simian immunodeficiency virus (SIV), chimeric simian/human immunodeficiency virus (SHIV) and related viruses. Studies on such models have unraveled a previously underscored protective potential against *in vivo* immunodeficiency virus replication. Pre-challenge NAb titers feasibly provide sterile protection from SIV/SHIV infection by purging the earliest onset of viral replication and likely modulate innate immune cell responses. Sufficient sub-sterile NAb titers after established infection also confer dose-dependent reduction of viremia, and in certain earlier time frames augment adaptive immune cell responses and even provide rebound-free viral control. Here, we provide an overview of the obtained patterns of SIV/SHIV protection and viral control by various types of NAb passive immunizations and discuss how these notions may be extrapolated to NAb-based clinical control of HIV infection.

## Introduction

Viral infections overcoming the host sentinel hurdles of cell-intrinsic and innate immune responses are met with well-concerted adaptive immune responses. Adaptive immune responses comprise cellular and humoral effectors, and the central players for each are CD8^+^ cytotoxic T lymphocytes (CTLs) targeting infected cells and neutralizing antibodies (NAbs) targeting viral envelopes (Envs). Normally, a combinational response of these two effectors, initiated and assisted by professional antigen-presenting cells (APCs) such as dendritic cells (DCs) and CD4^+^ helper T lymphocytes (Th), effectively kill and neutralize infected cells and cell-free virus, respectively, resulting in elimination of virus from the infected host.

**Figure 1:**
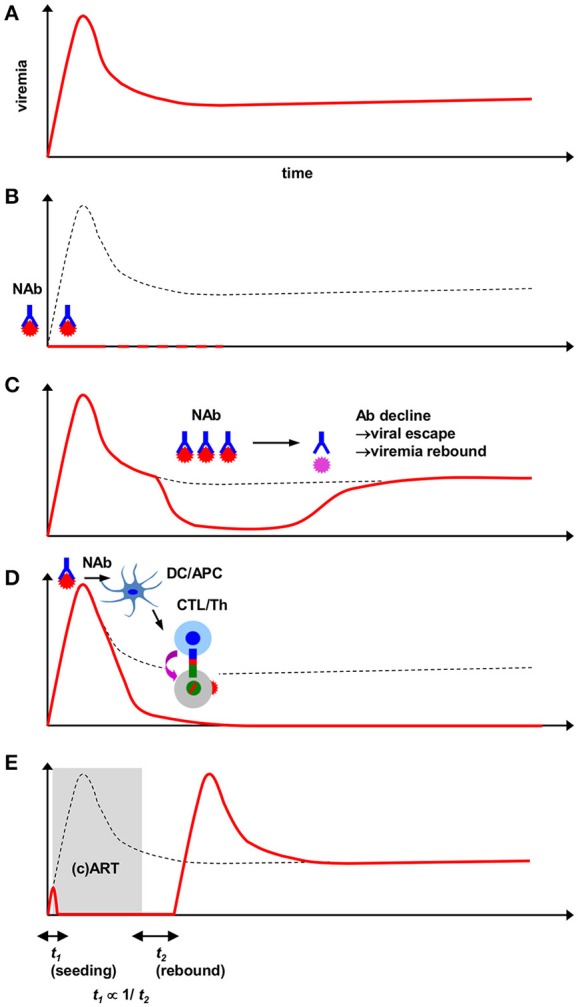
**Viremia patterns in HIV/SIV/SHIV-infected animals receiving interventions including passive NAbs. (A)** Course of persistent viremia in naïve infection. **(B)** Sterile protection against HIV/SIV/SHIV challenge by pre-challenge NAbs. Depending on the type of antibody, anti-HIV bNAbs can provide such protection at modest serum titers. Superacute-phase (1–10d) NAbs similarly mediate elimination of viral reservoirs. Complete elimination of virus (dotted lines) may be evaluated by experimental interventions such as CD8^+^-cell depletion. **(C)** Viremia reduction by subacute-phase post-challenge NAbs. An antibody dose-dependent attainment of viral load reduction (generally 10–1000-fold) is observed. Pharmacological decline of NAb titers and subsequent epitope-specific viral NAb escape results in rebound of viremia. **(D)** Early passive NAb-mediated sustained SIV viremia control by augmented virus-specific T-cell responses. NAb administration near peak viremia provides viral accumulation in DCs and functional augmentation in CTLs(/Th). Depending on combination of host/virus, B-cell responses are alternatively augmented. Neutralizing activity is critical for this modulation. **(E)** Antiretroviral therapy-based transient suppression of SIV viremia for comparison with NAb-based viremia suppression. Early *in vivo* viral dissemination (proportionate to *t*_1_) inversely correlates with the time to post-therapy cessation viremia rebound (*t*_2_). Judged from the uniform outcome of viral rebound, antiretroviral therapy likely does not modulate endogenous host immune responses.

Unfitting such an optimal course, CCR5^+^ (R5) memory CD4^+^ T cell-tropic (R5-tropic) human immunodeficiency virus (HIV) and pathogenic simian immunodeficiency virus (SIV) infections are met with inefficient adaptive immune responses, resulting in persistent viral replication (Figure 1A). CTL responses play a still incomplete yet central role in primary resolution of viremia (Goulder and Watkins, 2008), whereas there is a more major impairment in early NAb responses in typical HIV/SIV infections (Tomaras et al., 2008). Delayed HIV/SIV-specific NAb induction also accompanies very distinct traits, contrasting other viral infections; firstly, germinal center formation itself is delayed for more than 1 month (Levesque et al., 2009; Peruchon et al., 2009). Approximately past 3 months post-infection NAbs appear, which is severely delayed, and they repetitively succumb to viral escape (Richman et al., 2003). Along this course of NAb development, cross-reactivity against other HIV strains is gradually acquired by NAbs at about year 1 post-infection (Mikell et al., 2011). Later on, certain rare patients further proceed to eliciting NAbs showing very extensive cross-reactivity, which became defined as broadly neutralizing antibodies (bNAbs) (Burton et al., 1994). These bNAbs can now be identified and characterized by single-cell B-cell receptor cloning (Scheid et al., 2009).

NAb absence in early infection conversely suggests the importance of identifying anti-HIV antibody defense mechanisms as well as induction strategies. A practical approach for mechanistically analyzing NAb-based HIV control is antibody passive immunization. Here, recent progress on antibody passive immunization experiments in several types of animal AIDS models will be discussed (Table 1).

**Table 1:** **Anti-HIV/SIV antibody passive immunization in CCR5-tropic immunodeficiency virus-infected animals^**a**^**.

**Notion**	**Ab administration**	**Ab name/property^b^**	**Dosing**	**Host**	**Challenge Route/Virus^c^**	**Outcome**	**References**
Blockade of initial virus replication	Pre-challenge	b12	25 mg/kg	RM (Rhesus Macaque)	IVa SHIV_SF162P4_	First model of bNAb sterile protection against CCR5-tropic AIDS virus	Parren et al., 2001
	Pre-challenge	b12	25 mg/kg	RM	IVa SHIV_SF162P3_	Fc receptor-dependent bNAb sterile protection	Hessell et al., 2007
	Pre-challenge	VRC01/PGT121	20–50/0.2–20 mg/kg	RM	IR SHIV_AD8EO_/SHIV_DH12−V3AD8_	bNAb sterile protection: serum titer ~1:100	Shingai et al., 2014
	Pre-challenge	PGT121	2 mg/kg	RM	IVa SHIV_SF162P3_	bNAb distal elimination of viral reservoir cells	Liu et al., 2016
	Pre-challenge	Weak NAb b6/ Non-NAb F240	5 mg	RM	IVa SHIV_SF162P4_	No sterile protection by weak NAbs/non-NAbs	Burton et al., 2011
	Pre-challenge	Anti-HIV non-neut IgG from elite controllers	50 mg/kg	RM	IR SHIV_SF162P3_	No sterile protection by non-NAbs despite high ADCC activity	Dugast et al., 2014
	Days 1+4+7+10	VRC07−523+PGT121	10/40 mg/kg	Infant RM	Oral SHIV_SF162P3_	Elimination of viral reservoir establishment	Hessell et al., 2016
Adaptive immune-cell enhancement and improved prognosis	Pre-challenge	Polyclonal anti-SHIV neut IgG+b12	200 mg/kg	Infant pigtail macaque	Oral SHIV_SF162P3_	Enhanced endogenous B-cell responses and delayed disease onset	Ng et al., 2010
	Pre-challenge	Polyclonal anti-SHIV neut IgG+b12	200 mg/kg	Infant RM	Oral SHIV_SF162P3_	Enhanced endogenous B-cell responses associated with lower set point viremia and longer survival	Jaworski et al., 2013
	Days 1+14	Polyclonal anti-SIV neut IgG	170 mg/kg	RM	IV SIV_smE660_	Cases of sterile protection/viremia reduction, enhanced B-cell responses	Haigwood et al., 2004
	Day 7	Polyclonal anti-SIV neut IgG	300 mg	RM	IV SIV_mac239_	Augmented endogenous CTL-based elite control (-yr 2)	Iseda et al., 2016
	Day 7	Polyclonal anti-SIV non-neut IgG	300 mg	RM	IV SIV_mac239_	No viremia reduction by non-NAbs despite ADCVI activity	Nakane et al., 2013
Dose-dependent viremia reduction	Days 16–22	3BC176+PG16+45−46^G54W^ +PGT128+10−1074	0.5 mg each	Humanized mice	IP HIV-1_YU2_	10–1000-fold decrease in viremia and delayed rebound	Klein et al., 2012
	Week 12/157–163	3BNC117/10−1074	10 mg/kg	RM	IR SHIV_AD8EO_	NAb dose-dependent viremia reduction	Shingai et al., 2013
	Month 9 (d0+7+222)	PGT121	10 mg/kg	RM	IR SHIV_SF162P3_	NAb dose-dependent viremia reduction/ chronic-phase T-cell functional recovery	Barouch et al., 2013
	Day 153/745	Polyclonal anti-SIV neut IgG	170 mg/kg	RM rapid progressors	IV SIV_mac251_	Transient (<24 h) viremia decrease	Binley et al., 2000

a *Slashes show differential regimens. Plus signs show combination of the regimens. Experiments using are shown in temporally descending order of (N)Ab infusion*.

b *Neut, neutralizing; non-neut, non-neutralizing*.

c *IVa, intravaginal; IR: intrarectal; IV, intravenous; IP: intraperitoneal challenge*.

## Sterile protection and viremia suppression by passive NAbs

The initial interest in the field of HIV antibodies was whether pre-challenge NAbs may actually provide sterile protection against the incoming virus. Early implications had been obtained in a report on SIV-challenged cynomolgus macaques (Putkonen et al., 1991), while viral quantitation (and thus evaluation of protectivity) was relatively suboptimal at that time. Later work demonstrated NAb sterile protection against CXCR4-tropic (X4-tropic) HIV in human lymphocyte-reconstituted immunodeficient mice (Gauduin et al., 1997) and X4-tropic SHIV (X4-SHIV) challenge in pigtail macaques (Shibata et al., 1999) and rhesus macaques (Mascola et al., 1999, 2000; Baba et al., 2000). Further analysis on X4-SHIV models proposed theoretical requisite NAb titers (Nishimura et al., 2002) and also showed that the temporal window period permitting NAb-mediated sterile protection was very stringent (<24 h) (Nishimura et al., 2003).

Next addressed was the fundamental question on how NAbs may protect against the more difficult-to-protect CCR5-tropic (R5-tropic) immunodeficiency viruses which propagate more dominantly *in vivo* in the acute phase as transmitted/founder strains (Keele et al., 2008). An early challenging work (Parren et al., 2001) showed that, as like against X4-SHIVs, bNAb b12 with sufficient titers can exert complete protection against R5-tropic SHIV (R5-SHIV) challenge (Table 1 and Figure 1B). This concept of R5-tropic virus sterile protection was further confirmed by several combinations of bNAbs and viral challenge routes (Veazey et al., 2003; Hessell et al., 2009a,b, 2010). While it was first speculated that attainment of such sterile protection-conferring titers was rather difficult, recent studies have suggested that depending on the choice and potency of the passive bNAb, sterile protection may well be a feasible goal (Rudicell et al., 2014; Shingai et al., 2014). Another related new study has discovered that the biological half-life of the bNAbs determine the longevity of sterile protection against repeated SHIV challenges afforded by a single administration (Gautam et al., 2016). Based on the robust HIV/SIV-blocking efficacy *in vitro* as well as theoretically maximal “breadth,” interest had also been taken in blocking the CD4 entry receptor itself by antibodies, while recently it was shown that straightforwardly blocking the viral Env by bNAbs was more effective (Pegu et al., 2014).

Alternatively, it was also found that polyclonal neutralizing IgG existing at viral challenge can confer slower disease onset as well as enhanced survival in orally R5-SHIV-infected newborn macaques, suggestive of a lasting protective effect by preexisting NAb titers (Ng et al., 2010; Jaworski et al., 2013).

Extended from these, recently it was found that even in established chronic-phase R5-SHIV infection (post-set point), potent anti-HIV bNAbs at high titers can also provide viremia suppression (Barouch et al., 2013; Shingai et al., 2013) (Table 1 and Figure 1C). In such models, plasma bNAb titers, viral suppression and rebound basically are linked in a pharmacological manner; i.e., decline in NAb titers results in viral escape and viremia recrudescence. Furthermore, a recent report of HIV viremia suppression in stably infected (~20 days) humanized mice (Klein et al., 2012) (Table 1) showed that, compared with bNAb monotherapy, tri-mix or penta-mix administration of bNAbs resulted in a much sharper decline in viremia as well as delay in viremia rebound; i.e., an increase in the number of simultaneous Env targeting resulted in additive and/or synergistic containment of viral replication. This result also provided a rationale of inducing and/or administering multiple epitope-specific (b) NAbs for maximal efficacy. Thus, pre-challenge NAb titers do provide sterile protection against AIDS virus challenge and viremia suppression can also be transiently attained by sufficient post-challenge NAb titers.

## Innate immune-cell boosting by passive NAbs

How passive Ab administration may affect endogenous immune responses has been a long-standing important question. In the report on R5-SHIV sterile protection by b12 (Parren et al., 2001), the authors noted that NAbs, at sub-sterilizing titers, also derived cases of decreased viremia levels later on. Later, the group showed that the b12 sterile protection occurred *in vivo* in an Fc receptor- but not complement-dependent manner (Hessell et al., 2007), and associated with antibody-dependent cellular viral inhibition (ADCVI) (Forthal et al., 2001) activity *in vitro*. These innate (mainly natural killer) cell-dependent mechanisms were also explanatory of the above non-sterile protective effects. Another line of work showed engineered Fc receptor-binding properties of bNAb VRC01 being related with extended bioavailability, altered localization (antibody transcytosis) and improved protection against SHIV challenge (Ko et al., 2014). Recently, treatment-naïve HIV-infected patients manifested evidence for selective pressure by antibody-dependent cellular cytotoxicity (ADCC) even without exogenous Ab infusion (Chung et al., 2011), suggesting that augmenting this innate cell-dependent molecular/cellular axis may indeed be a promising strategy. Indeed, in HIV-1-infected humanized mice, enhanced binding against activating Fc receptors increased while its complete abrogation notably decreased bNAb-mediated viremia suppression (Bournazos et al., 2014), suggesting that innate cell modulation may also occur in established infection.

## Adaptive immune-cell boosting by passive NAbs

Intimately linked with the above is how NAbs may influence adaptive cellular responses. In our SIV challenge-NAb passive immunization model, we found that early (day 7) passive polyclonal NAb infusion in SIV_mac239_-challenged rhesus macaques resulted in elevated myeloid DC-associated viral loads (Yamamoto et al., 2007), temporally followed by elevation of Gag-specific polyfunctional CD4^+^ T-cell responses and increased *in vitro* viral suppressive activity in CD8^+^ cells (Yamamoto et al., 2009) (Table 1 and Figure 1D). Extended from such findings, in this model we recently identified that these NAb-mediated CD8^+^ cells also acquired enhanced suppressive activity against a panel of immunodominant CTL escape mutants, providing stringent T cell-based SIV control for up to 2 years without accumulation of viral CTL escape mutations (Iseda et al., 2016). This poses a possibility that the total virus-specific CTL population in NAb-infused animals became resistant against arousal of SIVs with CTL escape mutations *in vivo*, contributing to the prevention of CTL escape mutation accumulation. This early CTL “functional broadening” in NAb-infused macaques also may be related with the identified direct DC-mediated CTL cross-priming activity of the infused NAbs, suggestive of Ab/APC-dependent epitope spreading. Upon attainment of this stable SIV control, the originally immunodominant epitope-specific CTLs became preserved (presumably due to early CTL broadening) and showed stimuli-specific metabolic quiescence, as defined by enrichment of a phosphorylated AMP kinase-low CTL subpopulation which is indicative of exhaustion-free T-cell qualitative preservation (Blagih et al., 2015). Thus, NAb-boosted T cell-based primary SIV control also secondarily results in functional preservation of the most potent CTLs, which in turn may have further stabilized the viral control.

The above results collectively well explained SIV control in the animals, i.e., by passive NAb-mediated improved acute-phase CD4^+^/CD8^+^ T-cell priming. Alternatively, in another two-dose (days 1 plus 14) polyclonal NAb infusion model, cases of sterile protection and set-point viremia reduction were obtained in SIV_smE660_-challenged macaques, and this associated with enhanced endogenous *de novo* NAb responses (Haigwood et al., 1996, 2004). These similar phenotypes triggering different endogenous immune effector responses may stem from the different properties of the challenge virus strain (i.e., SIV_mac239_ is highly NAb-resistant and induces NAb responses only rarely and in the chronic phase). Importantly, this pattern of synergism between endogenous adaptive immune cells and early short-term NAb administration has also become well-conceptualized in murine retrovirus-infected mice (Gros et al., 2008; Michaud et al., 2010; Nasser et al., 2010), further emphasizing the importance of actively modifying T-cell and/or B-cell responses through coexisting NAbs.

In the aforementioned chronic-phase SHIV viremia suppression study (Barouch et al., 2013), bNAb infusion in macaques similarly provided an increase in CTL viral suppressive activity. Given that virus-specific CTLs show considerable functional exhaustion due to antigen load in chronic infection (Streeck et al., 2008), this post-NAb infusion increase in viral suppression may reflect a functional recovery, and hence may also involve viremia decrease itself in the recovery process.

Another well-designed AIDS model utilizes infant macaques to evaluate the direct impact of NAbs on impeding pathogenic progression with a clear-cut disease phenotype. Here, pre-challenge polyclonal and monoclonal NAbs conferred enhanced endogenous B-cell responses against oral R5-SHIV challenge and protection against disease onset (Ng et al., 2010; Jaworski et al., 2013). This may be a consequence of CD4^+^ T-cell protection, or due to some pattern of indirect B-cell modulation, such as NAb-mediated Env antigenic modulation (Schoofs et al., 2016). Conversely, late-phase polyclonal NAb infusion in immunocompromised SIV-infected rapid progressors showed no protective effect (Binley et al., 2000), suggesting the importance of intact endogenous immunity for NAb-mediated viral suppression. Taken together, results collectively show that NAbs provide a wide spectrum of protective mechanisms *in vivo*, particularly those involving Fc receptors (Lambour et al., 2016), against AIDS virus replication.

## Comparison between passive NAbs and drug therapy

How passive NAbs, which indeed are soluble effectors, may be pharmacologically compared for its impact with (combined) antiretroviral therapy [(c)ART] is another important point. One work on the effect of acute-phase ART on SIV-infected rhesus macaques (Kubo et al., 2009) showed that initiation of ART as early as day 2 (and up to day 28) still does not eliminate *in vivo* virus, as demonstrated by rapid rebound of viremia. Another further systematic analysis on temporal establishment of *in vivo* viral reservoir seeding showed that past day 3 post-SIV challenge, the time until initiation of cART is proportionate to the speed of viremia rebound after therapy cessation (Whitney et al., 2014) (Figure 1E). This implicates that the acknowledged notion of “hit early and hard” for drug therapy (Ho, 1995) also partially applies to the superacute phase, while attainment of complete viral eradication by ART is still an independent and extremely high final hurdle. This potentially may require host dispositions different from ones associated with elite HIV control such as possession of protective major histocompatibility complex class I (MHC-I) alleles, as implicated in the VISCONTI study in which early cART-treated patients with no viral rebound did not possess them (Sáez-Cirión et al., 2013).

In stark contrast, a recent important report showed that superacute-phase (days 1–10) administration of bNAbs (Hessell et al., 2016) results in an elimination in virus-detectable tissue compartments throughout the body for 6 months. Another cutting-edge report (Liu et al., 2016) tracked previously underscored post-infection viral replication *in situ* deriving transcriptomic signatures of antiviral gene up-regulation in infected foci (Barouch et al., 2016), and its abortion by *in vivo* titers of bNAb PGT121 at viral challenge. These two essentially may highlight a common notion of literally purging the eclipse phase of initial viral infection/dissemination by NAbs. It remains to be clarified whether the slightly earlier moment of NAb infusion derives the protection not obtained by early cART, or if other effector functions of NAbs unavailable by drugs mediate the protective effect. Our model of an acute-phase single NAb infusion resulting in sustained SIV control (Iseda et al., 2016) is most suggestive for the latter. Implications are also provided by a report showing that a combined bNAb single shot at day 10 post-SHIV infection derives protective effects substituting and comparable to that of a daily ART regimen for the next 11 days (Bolton et al., 2016). Taken together, NAbs do appear to exert protective effects unavailable by antiretroviral therapy.

## Requisite of neutralizing activity in passive antibody-based viral prevention and control

Whether direct virus-neutralizing activity is required for antibody-mediated AIDS virus control is critical. A very important report showed that for attaining stringent sterile protection against R5-SHIV challenge, neutralizing activity is indispensable (Burton et al., 2011). This notion was confirmed by another group, which showed that regardless of possessing potent Fc effector functions [ADCC and antibody-dependent cellular phagocytosis (ADCP) (Pelegrin et al., 2015)], antibody sterile protection was not obtained by pre-infection non-NAbs (Dugast et al., 2014) (Table 1). Similarly, in acute-phase infection, polyclonal non-NAb infusion at day 7 achieved no SIV viremia control in our model (Nakane et al., 2013). We very recently compared viral suppressive activity in our aforementioned polyclonal NAb- and non-NAb-infused rhesus macaques, and found that CD8^+^-cell viral suppressive activity is selectively enhanced in NAb-infused but not in non-NAb-infused animals (Yamamoto et al., 2016). This means that in addition to the availability of direct virus neutralization, such property of antiviral antibodies may also directly affect modulation patterns of cellular immune responses and further impact disease prognosis.

## Passive NAb immunotherapy in HIV-infected humans

Based on recent characterization of various bNAbs, their application as immunotherapeutic agents has started to extensively proceed in human trials. Preceding this current trend, insights had been obtained by one early clinical trial (Trkola et al., 2005), which showed that earlier administration of bNAbs against HIV-infected humans resulted in more delayed viremia rebound in several patients. In addition to considerations on such endogenous baseline status of the patients to be treated, another current interest is how the previously monitored association of virological control and (b) NAb pharmacological properties can be extrapolated from mice and non-human primates to humans.

In one representative study, single administration of a well-characterized potent CD4 binding site-specific bNAb VRC01 to HIV-infected patients was reported to exert 1.1–1.8 log10 reduction in viremia (Lynch et al., 2015). This single injection also derived emergence of VRC01-resistant strains, suggesting the necessity of either modifying or combining the NAbs to be infused.

Administration of another extremely potent CD4 binding site-specific bNAb 3BNC117, at a modest dose (30 mg/kg), also resulted in significant 0.8–2.5 log10 reduction in viremia (Caskey et al., 2015). 3BNC117 administration in HIV-infected humans also resulted in suppression of viral rebound during cART interruption (Scheid et al., 2016), highlighting the feasibility of and strong selective pressure exerted by this bNAb infusion. Furthermore, 3BNC117 administration in HIV-infected viremic individuals resulted in altered endogenous B-cell responses, as analyzed by changes in viral Env and B-cell receptor phylogenetic polymorphisms (Schoofs et al., 2016).

In another cohort, administration of an Env variable region 3 (V3)-specific bNAb KD-247 resulted in decreased viremia in HIV-infected patients, as well as an obtained case of ongoing viremia control after NAb decline (Matsushita et al., 2015), showing that a V3-specific bNAb can also mediate viral suppression. This bNAb further holds promise for host protection in that it may give synergistic protective effects with CD4-mimetic chemical compounds (Yoshimura et al., 2010). This and other types of NAb/chemical compound synergisms (Yoshimura et al., 2014; Madani et al., 2016) may become important strategies, particularly in NAb-based immunotherapies.

Collectively, these reports are now starting to provide a proof-of-concept for the notions initially obtained in animal AIDS models of NAb passive immunization.

## Concluding remarks

NAbs, when present by passive immunization, are being recognized as capable of playing a central role in sterile protection against and post-infection control of SIV and SHIV infection. Future studies aiming for NAb immunotherapy-based HIV clinical control shall proceed with a pharmacological perspective on the *in vivo* spectrum/dosage as well as Fc-mediated effector functions of the (b) NAbs to be infused. For prophylactic vaccine induction of HIV-specific NAbs, in addition to the rational design of vaccine Env antigen, designing attainment of synergism with concomitantly induced T-cell responses may delineate protective responses much more potent than what is currently expected.

## Author contributions

HY conceived and drafted manuscript; TM co-drafted manuscript.

## Funding

This work was supported by Japan Agency for Medical Research and Development, the Ministry of Health, Labor, and Welfare and the Ministry of Education, Culture, Sports, Science, and Technology in Japan ([JSPS] KAKENHI).

### Conflict of interest statement

The authors declare that the research was conducted in the absence of any commercial or financial relationships that could be construed as a potential conflict of interest.

## References

[B1] Baba T. W. Liska V. Hofmann-Lehmann R. Vlasak J. Xu W. Ayehunie S.. (2000). Human neutralizing monoclonal antibodies of the IgG1 subtype protect against mucosal simian-human immunodeficiency virus infection. Nat. Med. 6, 200–206. 10.1038/72309 10655110

[B2] Barouch D. H. Ghneim K. Bosche W. J. Li Y. Berkemeier B. Hull M.. (2016). Rapid inflammasome activation following mucosal SIV infection of Rhesus monkeys. Cell 165, 656–667. 10.1016/j.cell.2016.03.021 27085913 PMC4842119

[B3] Barouch D. H. Whitney J. B. Moldt B. Klein F. Oliveira T. Y. Liu J.. (2013). Therapeutic efficacy of potent neutralizing HIV-1-specific monoclonal antibodies in SHIV-infected rhesus monkeys. Nature 503, 224–228. 10.1038/nature12744 24172905 PMC4017780

[B4] Binley J. M. Clas B. Gettie A. Vesanen M. Montefiori D. C. Sawyer L.. (2000). Passive infusion of immune serum into simian immunodeficiency virus-infected rhesus macaques undergoing a rapid disease course has minimal effect on plasma viremia. Virology 270, 237–249. 10.1006/viro.2000.0254 10772996

[B5] Blagih J. Coulombe F. Vincent E. E. Dupuy F. Galicia-Vázquez G. Yurchenko E.. (2015). The energy sensor AMPK regulates T cell metabolic adaptation and effector responses *in vivo*. Immunity 42, 41–54. 10.1016/j.immuni.2014.12.030 25607458

[B6] Bolton D. L. Pegu A. Wang K. McGinnis K. Nason M. Foulds K.. (2016). Human immunodeficiency virus type 1 monoclonal antibodies suppress acute simian-human immunodeficiency virus viremia and limit seeding of cell-associated viral reservoirs. J. Virol. 90, 1321–1332. 10.1128/JVI.02454-15 26581981 PMC4719604

[B7] Bournazos S. Klein F. Pietzsch J. Seaman M. S. Nussenzweig M. C. Ravetch J. V. (2014). Broadly neutralizing anti-HIV-1 antibodies require Fc effector functions for *in vivo* activity. Cell 158, 1243–1253. 10.1016/j.cell.2014.08.023 25215485 PMC4167398

[B8] Burton D. R. Hessell A. J. Keele B. F. Klasse P. J. Ketas T. A. Moldt B.. (2011). Limited or no protection by weakly or nonneutralizing antibodies against vaginal SHIV challenge of macaques compared with a strongly neutralizing antibody. Proc. Natl. Acad. Sci. U.S.A. 108, 11181–11186. 10.1073/pnas.1103012108 21690411 PMC3131343

[B9] Burton D. R. Pyati J. Koduri R. Sharp S. J. Thornton G. B. Parren P. W.. (1994). Efficient neutralization of primary isolates of HIV-1 by a recombinant human monoclonal antibody. Science 266, 1024–1027. 10.1126/science.7973652 7973652

[B10] Caskey M. Klein F. Lorenzi J. C. Seaman M. S. West A. P. Jr. Buckley N.. (2015). Viraemia suppressed in HIV-1-infected humans by broadly neutralizing antibody 3BNC117. Nature 522, 487–491. 10.1038/nature14411 25855300 PMC4890714

[B11] Chung A. W. Isitman G. Navis M. Kramski M. Center R. J. Kent S. J.. (2011). Immune escape from HIV-specific antibody-dependent cellular cytotoxicity (ADCC) pressure. Proc. Natl. Acad. Sci. U.S.A. 108, 7505–7510. 10.1073/pnas.1016048108 21502492 PMC3088575

[B12] Dugast A. S. Chan Y. Hoffner M. Licht A. Nkolola J. Li H.. (2014). Lack of protection following passive transfer of polyclonal highly functional low-dose non-neutralizing antibodies. PLoS ONE 9:e97229. 10.1371/journal.pone.0097229 24820481 PMC4018276

[B13] Forthal D. N. Landucci G. Daar E. S. (2001). Antibody from patients with acute human immunodeficiency virus (HIV) infection inhibits primary strains of HIV type 1 in the presence of natural-killer effector cells. J. Virol. 75, 6953–6961. 10.1128/JVI.75.15.6953-6961.2001 11435575 PMC114423

[B14] Gauduin M. C. Parren P. W. Weir R. Barbas C. F. Burton D. R. Koup R. A. (1997). Passive immunization with a human monoclonal antibody protects hu-PBL-SCID mice against challenge by primary isolates of HIV-1. Nat. Med. 3, 1389–1393. 10.1038/nm1297-1389 9396610

[B15] Gautam R. Nishimura Y. Pegu A. Nason M. C. Klein F. Gazumyan A.. (2016). A single injection of anti-HIV-1 antibodies protects against repeated SHIV challenges. Nature 533, 105–109. 10.1038/nature17677 27120156 PMC5127204

[B16] Goulder P. J. R. Watkins D. I. (2008). Impact of MHC class I diversity on immune control of immunodeficiency virus replication. Nat. Rev. Immunol. 8, 619–630. 10.1038/nri2357 18617886 PMC2963026

[B17] Gros L. Pelegrin M. Michaud H. A. Bianco S. Hernandez J. Jacquet C.. (2008). Endogenous cytotoxic T-cell response contributes to the long-term antiretroviral protection induced by a short period of antibody-based immunotherapy of neonatally infected mice. J. Virol. 82, 1339–1349. 10.1128/JVI.01970-07 18032505 PMC2224427

[B18] Haigwood N. L. Montefiori D. C. Sutton W. F. McClure J. Watson A. Voss G.. (2004). Passive immunotherapy in simian immunodeficiency virus-infected macaques accelerates the development of neutralizing antibodies. J. Virol. 78, 5983–5995. 10.1128/JVI.78.11.5983-5995.2004 15140996 PMC415787

[B19] Haigwood N. L. Watson A. Sutton W. F. McClure J. Lewis A. Ranchalis J.. (1996). Passive immune globulin therapy in the SIV/macaque model: early intervention can alter disease profile. Immunol. Lett. 51, 107–114. 10.1016/0165-2478(96)02563-1 8811353

[B20] Hessell A. J. Hangartner L. Hunter M. Havenith C. E. Beurskens F. J. Bakker J. M.. (2007). Fc receptor but not complement binding is important in antibody protection against HIV. Nature 449, 101–104. 10.1038/nature06106 17805298

[B21] Hessell A. J. Jaworski J. P. Epson E. Matsuda K. Pandey S. Kahl C.. (2016). Early short-term treatment with neutralizing human monoclonal antibodies halts SHIV infection in infant macaques. Nat. Med. 22, 362–368. 10.1038/nm.4063 26998834 PMC4983100

[B22] Hessell A. J. Poignard P. Hunter M. Hangartner L. Tehrani D. M. Bleeker W. K.. (2009b). Effective, low-titer antibody protection against low-dose repeated mucosal SHIV challenge in macaques. Nat. Med. 15, 951–954. 10.1038/nm.1974 19525965 PMC4334439

[B23] Hessell A. J. Rakasz E. G. Poignard P. Hangartner L. Landucci G. Forthal D. N.. (2009a). Broadly neutralizing human anti-HIV antibody 2G12 is effective in protection against mucosal SHIV challenge even at low serum neutralizing titers. PLoS Pathog. 5:e1000433. 10.1371/journal.ppat.1000433 19436712 PMC2674935

[B24] Hessell A. J. Rakasz E. G. Tehrani D. M. Huber M. Weisgrau K. L. Landucci G.. (2010). Broadly neutralizing monoclonal antibodies 2F5 and 4E10 directed against the human immunodeficiency virus type 1 gp41 membrane-proximal external region protect against mucosal challenge by simian-human immunodeficiency virus SHIV_Ba-L_. J. Virol. 84, 1302–1313. 10.1128/JVI.01272-09 19906907 PMC2812338

[B25] Ho D. D. (1995). Time to hit HIV, early and hard. N. Engl. J. Med. 333, 450–451. 10.1056/NEJM199508173330710 7616996

[B26] Iseda S. Takahashi N. Poplimont H. Nomura T. Seki S. Nakane T.. (2016). Biphasic CD8^+^ T-cell defense in simian immunodeficiency virus control by acute-phase passive neutralizing antibody immunization. J. Virol. 90, 6276–6290. 10.1128/JVI.00557-16 27122584 PMC4936138

[B27] Jaworski J. P. Kobie J. Brower Z. Malherbe D. C. Landucci G. Sutton W. F.. (2013). Neutralizing polyclonal IgG present during acute infection prevents rapid disease onset in simian-human immunodeficiency virus SHIV_SF162P3_-infected infant rhesus macaques. J. Virol. 87, 10447–10459. 10.1128/JVI.00049-13 23885083 PMC3807376

[B28] Keele B. F. Giorgi E. E. Salazar-Gonzalez J. F. Decker J. M. Pham K. T. Salazar M. G.. (2008). Identification and characterization of transmitted and early founder virus envelopes in primary HIV-1 infection. Proc. Natl. Acad. Sci. U.S.A. 105, 7552–7557. 10.1073/pnas.0802203105 18490657 PMC2387184

[B29] Klein F. Halper-Stromberg A. Horwitz J. A. Gruell H. Scheid J. F. Bournazos S.. (2012). HIV therapy by a combination of broadly neutralizing antibodies in humanized mice. Nature 492, 118–122. 10.1038/nature11604 23103874 PMC3809838

[B30] Ko S. Y. Pegu A. Rudicell R. S. Yang Z. Y. Joyce M. G. Chen X.. (2014). Enhanced neonatal Fc receptor function improves protection against primate SHIV infection. Nature 514, 642–645. 10.1038/nature13612 25119033 PMC4433741

[B31] Kubo M. Nishimura Y. Shingai M. Lee W. Brenchley J. Lafont B.. (2009). Initiation of antiretroviral therapy 48 h after infection with simian immunodeficiency virus potently suppresses acute-phase viremia and blocks the massive loss of memory CD4^+^ T cells but fails to prevent disease. J. Virol. 83, 7099–7108. 10.1128/JVI.02522-08 19420078 PMC2704794

[B32] Lambour J. Naranjo-Gomez M. Piechaczyk M. Pelegrin M. (2016). Converting monoclonal antibody-based immunotherapies from passive to active: bringing immune complexes into play. Emerg. Microbes Infect. 5:e92. 10.1038/emi.2016.97 27530750 PMC5034104

[B33] Levesque M. C. Moody M. A. Hwang K. K. Marshall D. J. Whitesides J. F. Amos J. D.. (2009). Polyclonal B cell differentiation and loss of gastrointestinal tract germinal centers in the earliest stages of HIV-1 infection. PLoS Med. 6:e1000107. 10.1371/journal.pmed.1000107 19582166 PMC2702159

[B34] Liu J. Ghneim K. Sok D. Bosche W. J. Li Y. Chipriano E.. (2016). Antibody-mediated protection against SHIV challenge includes systemic clearance of distal virus. Science 353, 1045–1049. 10.1126/science.aag0491 27540005 PMC5237379

[B35] Lynch R. M. Boritz E. Coates E. E. DeZure A. Madden P. Costner P.. (2015). Virologic effects of broadly neutralizing antibody VRC01 administration during chronic HIV-1 infection. Sci. Transl. Med. 7:319ra206. 10.1126/scitranslmed.aad5752 26702094 PMC12366723

[B36] Madani N. Princiotto A. M. Easterhoff D. Bradley T. Luo K. Williams W. B.. (2016). Antibodies elicited by multiple envelope glycoprotein immunogens in primates neutralize primary human immunodeficiency viruses (HIV-1) sensitized by CD4-mimetic compounds. J. Virol. 90, 5031–5046. 10.1128/JVI.03211-15 26962221 PMC4859707

[B37] Mascola J. R. Lewis M. G. Stiegler G. Harris D. VanCott T. C. Hayes D.. (1999). Protection of Macaques against pathogenic simian/human immunodeficiency virus 89.6PD by passive transfer of neutralizing antibodies. J. Virol. 73, 4009–4018. 10196297 10.1128/jvi.73.5.4009-4018.1999 PMC104180

[B38] Mascola J. R. Stiegler G. VanCott T. C. Katinger H. Carpenter C. B. Hanson C. E.. (2000). Protection of macaques against vaginal transmission of a pathogenic HIV-1/SIV chimeric virus by passive infusion of neutralizing antibodies. Nat. Med. 6, 207–210. 10.1038/72318 10655111

[B39] Matsushita S. Yoshimura K. Ramirez K. P. Pisupati J. Murakami T. KD-1002 Study Group (2015). Passive transfer of neutralizing mAb KD-247 reduces plasma viral load in patients chronically infected with HIV-1. AIDS 29, 453–462. 10.1097/QAD.0000000000000570 25630040

[B40] Michaud H. A. Gomard T. Gros L. Thiolon K. Nasser R. Jacquet C.. (2010). A crucial role for infected-cell/antibody immune complexes in the enhancement of endogenous antiviral immunity by short passive immunotherapy. PLoS Pathog. 6:e1000948. 10.1371/journal.ppat.1000948 20548955 PMC2883599

[B41] Mikell I. Sather D. N. Kalams S. A. Altfeld M. Alter G. Stamatatos L. (2011). Characteristics of the earliest cross-neutralizing antibody response to HIV-1. PLoS Pathog. 7:e1001251. 10.1371/journal.ppat.1001251 21249232 PMC3020924

[B42] Nakane T. Nomura T. Shi S. Nakamura M. Naruse T. K. Kimura A.. (2013). Limited impact of passive non-neutralizing antibody immunization in acute SIV infection on viremia control in rhesus macaques. PLoS ONE 8:e73453. 10.1371/journal.pone.0073453 24039947 PMC3767751

[B43] Nasser R. Pelegrin M. Michaud H. A. Plays M. Piechaczyk M. Gros L. (2010). Long-lasting protective antiviral immunity induced by passive immunotherapies requires both neutralizing and effector functions of the administered monoclonal antibody. J. Virol. 84, 10169–10181. 10.1128/JVI.00568-10 20610721 PMC2937798

[B44] Ng C. T. Jaworski J. P. Jayaraman P. Sutton W. F. Delio P. Kuller L.. (2010). Passive neutralizing antibody controls SHIV viremia and enhances B cell responses in infant macaques. Nat. Med. 16, 1117–1119. 10.1038/nm.2233 20890292 PMC2952052

[B45] Nishimura Y. Igarashi T. Haigwood N. L. Sadjadpour R. Donau O. K. Buckler C.. (2003). Transfer of neutralizing IgG to macaques 6 h but not 24 h after SHIV infection confers sterilizing protection: implications for HIV-1 vaccine development. Proc. Natl. Acad. Sci. U.S.A. 100, 15131–15136. 10.1073/pnas.2436476100 14627745 PMC299920

[B46] Nishimura Y. Igarashi T. Haigwood N. Sadjadpour R. Plishka R. J. Buckler-White A.. (2002). Determination of a statistically valid neutralization titer in plasma that confers protection against simian-human immunodeficiency virus challenge following passive transfer of high-titered neutralizing antibodies. J. Virol. 76, 2123–2130. 10.1128/jvi.76.5.2123-2130.2002 11836389 PMC153825

[B47] Parren P. W. Marx P. A. Hessell A. J. Luckay A. Harouse J. Cheng-Mayer C.. (2001). Antibody protects macaques against vaginal challenge with a pathogenic R5 simian/human immunodeficiency virus at serum levels giving complete neutralization *in vitro*. J. Virol. 75, 8340–8347. 10.1128/JVI.75.17.8340-8347.2001 11483779 PMC115078

[B48] Pegu A. Yang Z. Y. Boyington J. C. Wu L. Ko S. Y. Schmidt S. D.. (2014). Neutralizing antibodies to HIV-1 envelope protect more effectively *in vivo* than those to the CD4 receptor. Sci. Transl. Med. 6:243ra88. 10.1126/scitranslmed.3008992 24990883 PMC4562469

[B49] Pelegrin M. Naranjo-Gomez M. Piechaczyk M. (2015). Antiviral monoclonal antibodies: can they be more than simple neutralizing agents? Trends Microbiol. 23, 653–665. 10.1016/j.tim.2015.07.005 26433697 PMC7127033

[B50] Peruchon S. Chaoul N. Burelout C. Delache B. Brochard P. Laurent P.. (2009). Tissue-specific B-cell dysfunction and generalized memory B-cell loss during acute SIV infection. PLoS ONE 4:e5966. 10.1371/journal.pone.0005966 19543531 PMC2695011

[B51] Putkonen P. Thorstensson R. Ghavamzadeh L. Albert J. Hild K. Biberfeld G.. (1991). Prevention of HIV-2 and SIVsm infection by passive immunization in cynomolgus monkeys. Nature 352, 436–438. 10.1038/352436a0 1677743

[B52] Richman D. D. Wrin T. Little S. J. Petropoulos C. J. (2003). Rapid evolution of the neutralizing antibody response to HIV type 1 infection. Proc. Natl. Acad. Sci. U.S.A. 100, 4144–4149. 10.1073/pnas.0630530100 12644702 PMC153062

[B53] Rudicell R. S. Kwon Y. D. Ko S. Y. Pegu A. Louder M. K. Georgiev I. S.. (2014). Enhanced potency of a broadly neutralizing HIV-1 antibody *in vitro* improves protection against lentiviral infection *in vivo*. J. Virol. 88, 12669–12682. 10.1128/JVI.02213-14 25142607 PMC4248941

[B54] Sáez-Cirión A. Bacchus C. Hocqueloux L. Avettand-Fenoel V. Girault I. Lecuroux C.. (2013). Post-treatment HIV-1 controllers with a long-term virological remission after the interruption of early initiated antiretroviral therapy ANRS VISCONTI Study. PLoS Pathog. 9:e1003211. 10.1371/journal.ppat.1003211 23516360 PMC3597518

[B55] Scheid J. F. Horwitz J. A. Bar-On Y. Kreider E. F. Lu C. L. Lorenzi J. C.. (2016). HIV-1 antibody 3BNC117 suppresses viral rebound in humans during treatment interruption. Nature 535, 556–560. 10.1038/nature18929 27338952 PMC5034582

[B56] Scheid J. F. Mouquet H. Feldhahn N. Seaman M. S. Velinzon K. Pietzsch J.. (2009). Broad diversity of neutralizing antibodies isolated from memory B cells in HIV-infected individuals. Nature 458, 636–640. 10.1038/nature07930 19287373

[B57] Schoofs T. Klein F. Braunschweig M. Kreider E. F. Feldmann A. Nogueira L.. (2016). HIV-1 therapy with monoclonal antibody 3BNC117 elicits host immune responses against HIV-1. Science 352, 997–1001. 10.1126/science.aaf0972 27199429 PMC5151174

[B58] Shibata R. Igarashi T. Haigwood N. Buckler-White A. Ogert R. Ross W.. (1999). Neutralizing antibody directed against the HIV-1 envelope glycoprotein can completely block HIV-1/SIV chimeric virus infections of macaque monkeys. Nat. Med. 5, 204–210. 10.1038/5568 9930869

[B59] Shingai M. Donau O. K. Plishka R. J. Buckler-White A. Mascola J. R. Nabel G. J.. (2014). Passive transfer of modest titers of potent and broadly neutralizing anti-HIV monoclonal antibodies block SHIV infection in macaques. J. Exp. Med. 211, 2061–2074. 10.1084/jem.20132494 25155019 PMC4172223

[B60] Shingai M. Nishimura Y. Klein F. Mouquet H. Donau O. K. Plishka R.. (2013). Antibody-mediated immunotherapy of macaques chronically infected with SHIV suppresses viraemia. Nature 503, 277–280. 10.1038/nature12746 24172896 PMC4133787

[B61] Streeck H. Brumme Z. L. Anastario M. Cohen K. W. Jolin J. S. Meier A.. (2008). Antigen load and viral sequence diversification determine the functional profile of HIV-1-specific CD8^+^ T cells. PLoS Med. 5:e100. 10.1371/journal.pmed.0050100 18462013 PMC2365971

[B62] Tomaras G. D. Yates N. L. Liu P. Qin L. Fouda G. G. Chavez L. L.. (2008). Initial B-cell responses to transmitted human immunodeficiency virus type 1: virion-binding immunoglobulin M (IgM) and IgG antibodies followed by plasma anti-gp41 antibodies with ineffective control of initial viremia. J. Virol. 82, 12449–12463. 10.1128/JVI.01708-08 18842730 PMC2593361

[B63] Trkola A. Kuster H. Rusert P. Joos B. Fischer M. Leemann C.. (2005). Delay of HIV-1 rebound after cessation of antiretroviral therapy through passive transfer of human neutralizing antibodies. Nat. Med. 11, 615–622. 10.1038/nm1244 15880120

[B64] Veazey R. S. Shattock R. J. Pope M. Kirijan J. C. Jones J. Hu Q.. (2003). Prevention of virus transmission to macaque monkeys by a vaginally applied monoclonal antibody to HIV-1 gp120. Nat. Med. 9, 343–346. 10.1038/nm833 12579198

[B65] Whitney J. B. Hill A. L. Sanisetty S. Penaloza-MacMaster P. Liu J. Shetty M.. (2014). Rapid seeding of the viral reservoir prior to SIV viraemia in rhesus monkeys. Nature 512, 74–77. 10.1038/nature13594 25042999 PMC4126858

[B66] Yamamoto H. Iseda S. Nakane T. Nomura T. Takahashi N. Seki S.. (2016). Augmentation of anti-simian immunodeficiency virus activity in CD8^+^ cells by neutralizing but not nonneutralizing antibodies in the acute phase. AIDS 30, 2391–2394. 10.1097/QAD.0000000000001221 27603164

[B67] Yamamoto H. Kawada M. Takeda A. Igarashi H. Matano T. (2007). Post-infection immunodeficiency virus control by neutralizing antibodies. PLoS ONE 2:e540. 10.1371/journal.pone.0000540 17579714 PMC1890307

[B68] Yamamoto T. Iwamoto N. Yamamoto H. Tsukamoto T. Kuwano T. Takeda A.. (2009). Polyfunctional CD4^+^ T-cell induction in neutralizing antibody-triggered control of simian immunodeficiency virus infection. J. Virol. 83, 5514–5524. 10.1128/JVI.00145-09 19297503 PMC2681982

[B69] Yoshimura K. Harada S. Boonchawalit S. Kawanami Y. Matsushita S. (2014). Impact of maraviroc-resistant and low-CCR5-adapted mutations induced by *in vitro* passage on sensitivity to anti-envelope neutralizing antibodies. J. Gen. Virol. 95, 1816–1826. 10.1099/vir.0.062885-0 24795449

[B70] Yoshimura K. Harada S. Shibata J. Hatada M. Yamada Y. Ochiai C.. (2010). Enhanced exposure of human immunodeficiency virus type 1 primary isolate neutralization epitopes through binding of CD4 mimetic compounds. J. Virol. 84, 7558–7568. 10.1128/JVI.00227-10 20504942 PMC2897603

